# Towards automated cancer screening: Label‐free classification of fixed cell samples using wavelength modulated Raman spectroscopy

**DOI:** 10.1002/jbio.201700244

**Published:** 2018-01-30

**Authors:** Lana Woolford, Mingzhou Chen, Kishan Dholakia, C. Simon Herrington

**Affiliations:** ^1^ Edinburgh Cancer Research Centre, Institute of Genetics and Molecular Medicine University of Edinburgh Edinburgh UK; ^2^ SUPA, School of Physics and Astronomy University of St. Andrews Fife UK

**Keywords:** cancer, cervix, Raman spectroscopy, screening

## Abstract

The ability to provide quantitative, objective and automated pathological analysis would provide enormous benefits for national cancer screening programmes, in terms of both resource reduction and improved patient wellbeing. The move towards molecular pathology through spectroscopic methods shows great promise, but has been restricted by spectral quality, acquisition times and lack of direct clinical application. In this paper, we present the application of wavelength modulated Raman spectroscopy for the automated label‐ and fluorescence‐free classification of fixed squamous epithelial cells in suspension, such as those produced during a cervical smear test. Direct comparison with standard Raman spectroscopy shows marked improvement of sensitivity and specificity when considering both human papillomavirus (sensitivity +12.0%, specificity +5.3%) and transformation status (sensitivity +10.3%, specificity +11.1%). Studies on the impact of intracellular sampling location and storage effects suggest that wavelength modulated Raman spectroscopy is sufficiently robust to be used in fixed cell classification, but requires further investigations of potential sources of molecular variation in order to improve current clinical tools.

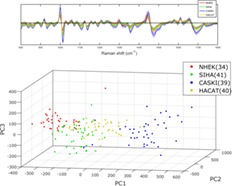

## 1 INTRODUCTION

The introduction of national screening programmes such as the Papanicoloau (‘Pap’) test and mammogram have achieved considerable success, and are estimated to prevent, for example, 4500 1 and 1300 2 deaths in the United Kingdom every year from cervical and breast cancer, respectively. However, these programmes require large, sustained financial input and human resources and are not completely accurate. The primary purpose of cervical screening is to detect abnormal epithelial cells that indicate the presence of cervical pre‐cancer or cancer. In a cervical screen, cells are brushed from the cervix and stored in fixing solution as a liquid‐based cytology sample. This ‘smear’ is transferred to a microscope slide, stained and viewed by a cytopathologist, who classifies it as normal, borderline, low or high grade dyskaryosis, glandular neoplasia or unsatisfactory 3. However, interpretation of cell morphology is hugely complex and therefore, open to subjectivity and false diagnosis, as small numbers of abnormal epithelial cells must be identified in a background of numerous normal epithelial and inflammatory cells. In 2008, clinical trial comparing reviewed with initial pre‐treatment histologic diagnosis, the probability of unnecessary treatment was found to be 8% to 27% (dependent on initial cytologic findings) 4, indicating that many women may be being referred to colposcopy unnecessarily. The use of biomarker screening has been posited as a potential method for improving diagnostic accuracy, but identifying targets relevant to genetically heterogeneous populations exposed to a variety of environmental factors while maintaining high‐positive predictive value is often unfeasible 5. Thus, a technique which provides the benefits of quantitative molecular pathology but with a broader scope and automated classification could vastly improve screening outcomes.

One technique with the potential to deliver these characteristics is Raman spectroscopy. Raman spectroscopy is a label‐free technique based on the inelastic scattering of light. It can be used to identify the molecular vibrational bonds present in a sample. Changes in disease state are accompanied by alterations in molecular pathology, which are reflected in spectral data. Raman spectroscopy in its various forms is emerging as a key contender for analysis of cancer status 6, including colorectal 7, lung 8 and cervical cancer 9, 10, 11. However, the likelihood of clinical uptake is highly dependent upon the ability to contend with the practicality as well as accuracy of diagnostic gold standards such as histopathology. As such, a new classification method would need to be incorporated into current systems for trials to be conducted in the first instance.

In this paper, we demonstrate the use of wavelength modulated Raman spectroscopy (WMRS) 12 as a tool for the automated classification of unlabeled fixed cells in suspension, reflecting its potential utility in diagnosing clinical liquid‐based cytology samples. One of the major technical barriers to the introduction of Raman spectroscopy as a clinical tool is the long acquisition time associated with the rare event of Raman scattering of photons. The acquisition of high‐quality spectra is further compounded by the presence of autofluorescence in samples, which is a particular problem for biological interrogation and can often lead to the loss of smaller Raman peaks obscured by a fluorescent background. Several approaches for reducing the background have been explored, including post‐acquisition baseline correction, nonlinear optical methods and time‐, frequency‐ and wavelength‐domain methods 13.

WMRS is a wavelength‐domain approach. During measurement, the laser wavelength is modulated throughout the acquisition process using a tunable laser 14. This causes the Raman spectral peaks to shift fractionally when measured in terms of scattered wavelength. When this shift is measured across several wavelengths, a ‘differential spectrum’ can be generated which demonstrates the true Raman peaks in wavenumber shift from the incident wavelength. Wavelength modulation does not affect the position of autofluorescence from the sample and substrate, allowing the background to be automatically removed 12. Methods for post‐processing wavelength modulated spectra in order to account for wavelength shift and remove this static fluorescence background have been tested, including standard excitation Raman difference spectroscopy, SD, Fourier filtering, least‐squares fit and principal component analysis (PCA), with PCA providing the best signal‐to‐noise ratio 15. The differential Raman spectrum is formed from the first principal component (PC) of the modulated data set, corresponding to the component which captures the greatest variance in the data.

This method has been used to investigate single living Chinese Hamster Ovary cells (CHO‐K1) 12 as well as T lymphocytes (CD4^+^, CD8^+^), killer T cells (CD56^+^) and dendritic cells (CD303^+^, CD1c^+^) 16. It has also been shown to provide high specificity and sensitivity for the characterisation of human urothelial (SV‐HUC‐1) and bladder cancer (MGH) cell lines cultured normally 14 or exposed to urine 17, which represents another step towards an automated optical platform for cancer screening. The aim of this study is to assess the practical potential of WMRS as a method for the automated classification of fixed squamous epithelial cells, modelling cervical cancer diagnosis.

## 2 MATERIALS AND METHODS

### 2.1 Cell sample preparation

**Figure 1:**
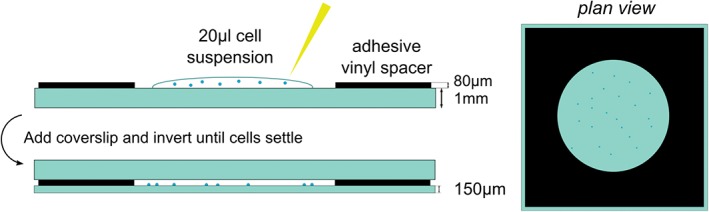
The cavity slide assembly used for all acquisitions. Slides were sterilised with ethanol, air‐dried and polished in between measurements

A total of 4 established cell lines, and primary cells, from squamous epithelial tissue were used for this project, reflecting the intention to investigate WMRS as a method of classifying cervical smear samples. CaSki, SiHa, C33A (all squamous cell carcinoma, adult human cervix), HaCaT (immortalised squamous epithelium, adult human skin) and primary normal human epidermal keratinocytes (‘NHEKs’, normal squamous epithelium, neonatal human skin) were cultured to 80% to 90% confluency. These were harvested by trypsinisation, washed in triplicate by centrifugation and resuspension in sterilised phosphate buffered saline (PBS), and fixed in the clinical methanol‐based fixative PreservCyt. Samples were stored in PreservCyt at −20^∘^C until use. All centrifugation steps were executed at 400. for 5 minutes at 4^∘^C. Prior to interrogation, fixed cell samples were washed in triplicate by centrifugation and resuspension in sterilised PBS in order to remove traces of fixing solution, as methanol produces strong Raman signatures. Twenty microliter of PBS cell suspension was then transferred to a quartz cavity slide (Figure 1), carefully sealed with a 150 μm quartz coverslip and inverted. The quartz assemblies were rested for at least 20 minutes prior to interrogation in order to promote cell adherence to the quartz coverslip, which was placed face down on the inverted microscope assembly for measurement. Details of the origin and cell culture reagents used for each cell type are provided in Appendix S1, Table 1.

### 2.2 Spectral acquisition

**Figure 2:**
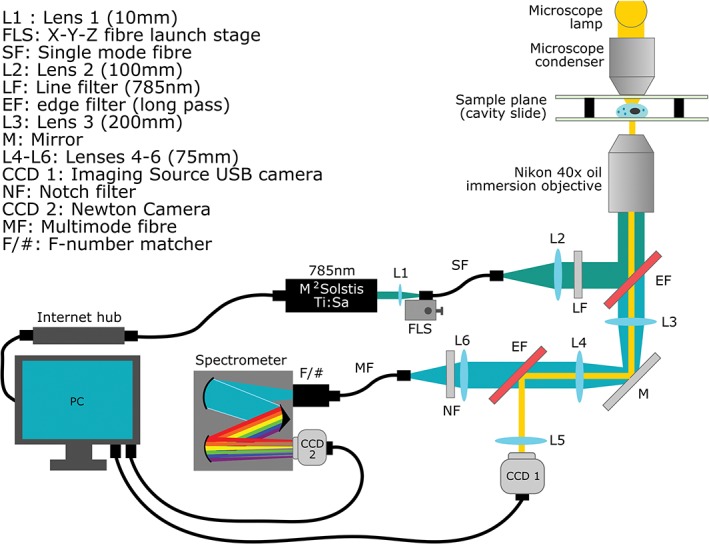
The optical diagram of the spectrometer designed for WMRS and standard Raman spectral acquisition. Brightfield microscope images are acquired separately on CCD1 and blocked from the spectrometer by secondary EF

A confocal Raman microscope built and modified for acquisition of both standard and modulated spectra (Figure 2) was used to evaluate the fixed cell samples. Raman spectra were generated from individual cells inside the quartz cavity assembly using a tunable continuous wave Ti‐Sapphire laser (‘SolsTis’, M Squared Laser Ltd, maximum power 1 W at 785 nm), delivering 150 mW power in a 1 μm spot in the sample plane through a 40× oil immersion objective (Nikon, NA1.3). A translational stage allowed the laser to be focused on the nuclear region of each cell, with Raman scattered photons collected from a confocal collection volume 2.86 μm in depth and 2.63 μm in diameter about the laser spot. The signal was resolved with a Shamrock SR‐303i spectrometer (400 lines/mm grating, Andor Technology) and collected with a CCD camera (Newton, Andor Technology) thermoelectrically cooled to −70^∘^C. ‘Standard’ spontaneous and wavelength modulated Raman spectra were acquired using Solis (s) Software (Andor Technology) along with a brightfield microscope image (Imaging Source USB Camera) of each cell (see Supplementary Information S3). Standard acquisitions were conducted at 785 nm, using 5 accumulations of 2 seconds laser exposure for both the cell and adjacent background. The adjacent background, including any spectral contribution from the quartz slide, was removed from the raw cell spectrum for data analysis. In the modulated acquisitions, 5 accumulations of 2 seconds exposures were performed with 5 different cycles of excitation wavelengths over a total range of Δ*λ* = 1 nm centred at 785 nm. No background acquisition was necessary here due to the inherent autofluorescence removal. It has been shown previously that no significant phototoxicity was experienced by the cells as a result of laser exposure at similar settings 16.

Reference spectra for laser calibration were acquired from 1 μm polystyrene beads in distilled water. These spectra were used to account for fluctuations in laser power, wavelength and spot location due to heating, fibre‐optic coupling or mirror alignment, as well as post‐acquisition spectral processing.

### 2.3 Spectral processing and data analysis

All spectra were considered in the fingerprint region of interest, 800 to 1800 cm^−1^. Spectra were normalised according to the total area under each modulation set, which is linearly proportional to the laser power. Laser wavelength was also monitored by linearly interpolating the intensity and Raman shift of the characteristic 1001.4 cm^−1^ polystyrene peak according to the sample measurement time stamp and adjusting spectral position accordingly. Overall, this accounts for any laser power and wavelength fluctuations over time, thereby preventing the PCA script from grouping cells according to these parameters. Immediately prior to analysis, individual spectra were assessed and removed from the analysis if a high spectral background was apparent. Such artefacts arise from suboptimal focusing or cell movement during acquisition. All spectra were analysed using PCA, a commonly used multivariate spectral analysis technique 18. PCA breaks spectra down into orthogonal spectral components PCs which describe the variation in the dataset. The first principal component describes the greatest variation, with higher components eventually describing noise 19. The first 7 PCs were used in all data analysis conducted. The weighting of these PCs (the extent to which each PC represents a particular spectrum in the dataset) was used to generate PCA cluster plots, which may be used for diagnostic classification. Sensitivity and specificity values were generated through ‘leave‐one‐out cross‐validation (LOOCV)’, also known as a ‘jacknife’ 18. When performed for 2 datasets, the classifications can be quantitatively estimated as sensitivity and specificity values by assigning the each cell line as ‘positive’ or ‘negative’. Statistically significant variations between spectra were also estimated for each cell line pair using student's . test at a significance level of . < 10^−12^. This was conducted using the established ‘ttest2’ function in MATLAB, where the average spectral intensity at each wavenumber is compared, with consideration to spectral variation across the dataset (indicated in figures by coloured bands around the average). The PCA script used was written in MATLAB by the Biophotonics Group at the University of St Andrews.

## 3 RESULTS

### 3.1 Comparison of human papillomavirus status

**Figure 3:**
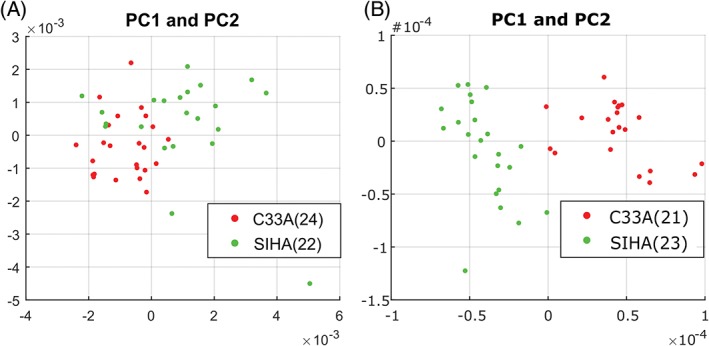
Standard (A) and modulated (B) spectral datapoints represented as PC1/2 weightings for C33A (HPV negative carcinoma) and SiHa (HPV‐16 carcinoma) cells. The number of cells used for each comparison are indicated in the legend

A preliminary comparison of standard and wavelength modulated Raman spectroscopy as a classification tool was made using fixed C33A human papillomavirus (HPV negative) and SiHa (HPV16 positive) cells, kindly donated by the Scottish HPV Archive 20. The samples were resuspended in PBS and prepared for analysis as described in Section 2.1. Initial analysis of the C33A cell line found that an acquisition time of 2 seconds over 5 accumulations yielded superior standard Raman spectra to a single acquisition of 10seconds. This is due to a comparative reduction in CCD camera noise for the shorter acquisitions. The brightfield cell images were morphologically similar, consisting of 10 μm cells which were highly rounded due to the trypsinisation process. The PCA plots for the standard and modulated Raman can be seen in Figure 3. LOOCV was applied to the datasets to yield sensitivity values of 88.0% and 100.0% for standard and modulated Raman, respectively, representing a considerable improvement of 12%. Specificities of 90.5% and 95.8% were found for standard and modulated Raman respectively, or a 5.3% improvement. It is clear from both PCA and LOOCV that modulated Raman spectroscopy provides a superior separation and classification of 2 cervical carcinoma cell lines with differing HPV status. The main spectral disparities occur at approximately 730, 760, 890, 920, 1130, 1250, 1380 and 1480 cm^−1^.

### 3.2 Comparison of different status in squamous epithelial cell lines

**Figure 4:**
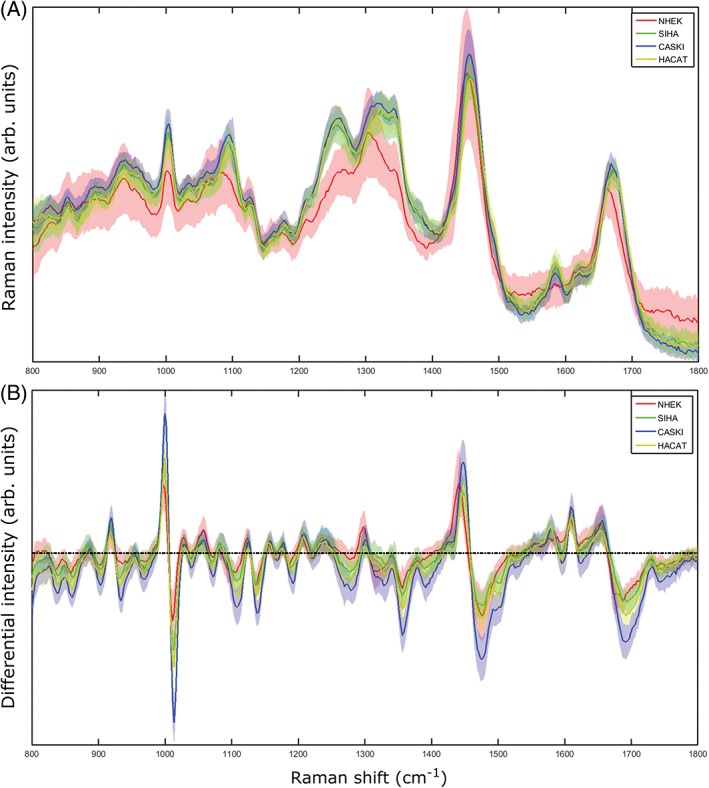
(A) Standard and (B) modulated Raman spectral averages for the 4 cell lines used, 800 to 1800 cm^−1^

**Figure 5:**
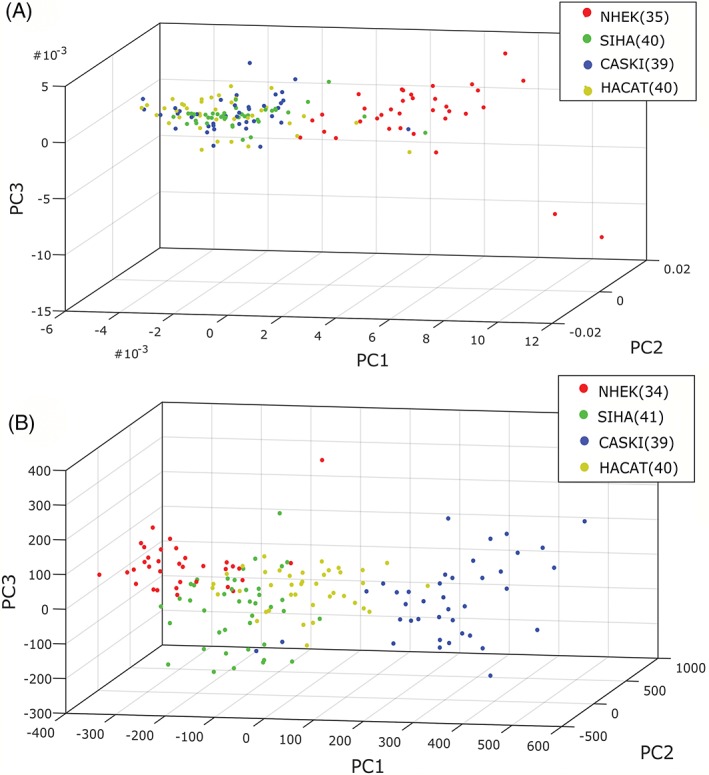
(A) Standard and (B) modulated PCA weightings for the 4 cell lines used. Variation in the number of datapoints used arises from the rejection of different spectra in each set

The investigation of standard and modulated Raman spectroscopy was then extended to 4 cell lines (1 normal, 1 immortalised and 2 transformed), which were chosen to represent the spectrum of abnormality typically found in the progression to cervical cancer. NHEKs are normal squamous epithelial cells which stop dividing when they reach a certain age. HaCaT cells 21 are a spontaneously immortalised epithelial line which can proliferate beyond this normal limit. These were used as a model for cervical precancer. SiHa 22 and CaSki 23 are cervical cancer cell lines from primary and metastatic tumours, respectively. These are both transformed phenotypes, consisting of cells capable of forming tumours. A total of 163 cells were interrogated using both standard and wavelength modulated Raman spectroscopy, consisting of 42 NHEK, 41 SiHa, 40 CaSki and 40 HaCaT cells. As before, the established cell lines in particular could not be discerned on a purely morphological basis, which furthers the case for biochemical classification as a complementary technique. It must be noted that the morphology of the primary NHEK cells varied significantly in terms of both shape and size compared to the established cell lines. Following removal of suboptimal spectra, Raman spectra and PCA plots were generated as seen in Figures 4 and 5, respectively. The ability of PCA to act as a classification algorithm was explored using cross‐validation of the data. LOOCV was performed on all 4 cell lines for both datasets; the confusion matrices for standard and modulated PCA can be seen in Table 1 and 2, respectively, along with misclassification statistics. For all 3 established cell lines, the modulated spectra provide better discrimination. However, classification is slightly superior using standard Raman spectra for NHEKs.

**Table 1:** The confusion matrix resulting from cross‐validation of standard Raman PCA, with accompanying percentage classification accuracy

Actual	Predicted				Class acc. (%)
	NHEK	SiHa	CaSki	HaCaT	
NHEK	31	2	1	1	88.6
SiHa	2	25	6	7	62.5
CaSki	1	9	19	10	48.7
HaCaT	3	9	6	22	55.0

**Table 2:** The confusion matrix resulting from cross‐validation of modulated Raman PCA, with accompanying percentage classification accuracy

Actual	Predicted				Class acc. (%)
	NHEK	SiHa	CaSki	HaCaT	
NHEK	29	4	0	1	85.3
SiHa	1	32	0	8	78.0
CaSki	0	1	38	0	97.4
HaCaT	0	2	0	38	95.0

**Figure 6:**
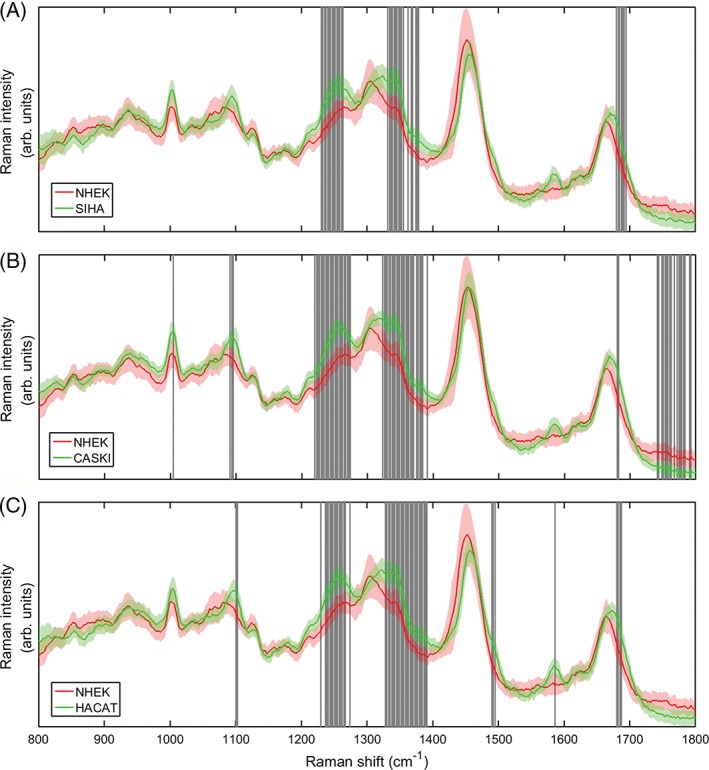
Direct comparison of the standard spectra for NHEK against the remaining established cell lines SiHa, CaSki and HaCaT. Shaded bands refer to regions of statisitically significant difference (student's . test) between the spectral intensities

**Figure 7:**
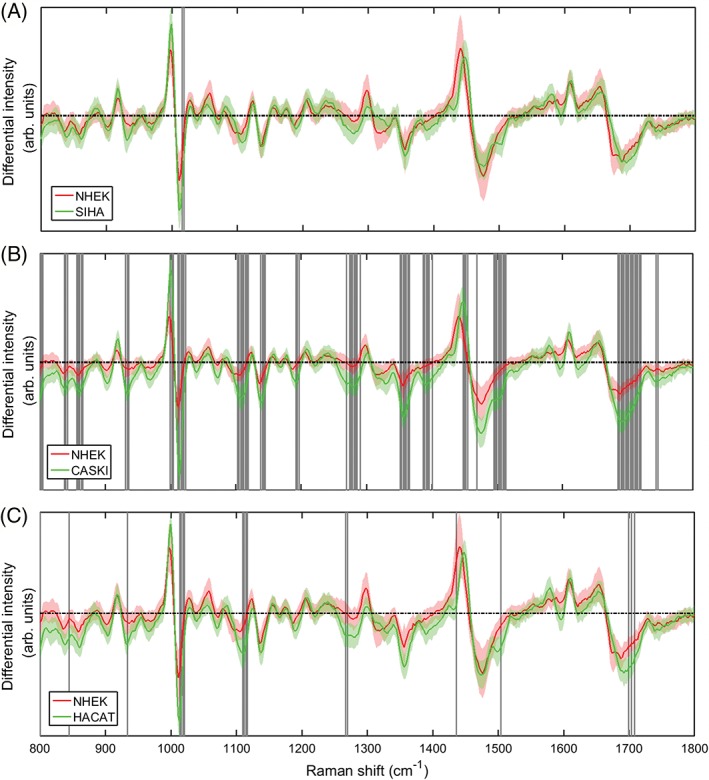
Direct comparison of the modulated spectra for NHEK against the remaining established cell lines SiHa, CaSki and HaCaT. Shaded bands refer to regions of statisitically significant difference (student's *t* test) between the spectral intensities

In order to gain further understanding of both the efficacy of the test and the major vibrational signals contributing to the analysis, each possible pair combination of cell lines was analysed using the PCA script. The average sensitivity was 84.8% ± 9.0. and 95.1% ± 5.6., and specificity was 85.1% ± 10.7. and 96.2% ± 4.6., for standard and modulated PCA respectively. This demonstrates that, for both metrics, wavelength modulated Raman spectroscopy provides superior discrimination (+10.3% sensitivity, +11.1% specificity) as well as lower variation between cell types. The generated spectra were also marked with significance bands as decribed in Section 2.3. Spectra with marked bands are shown for NHEK‐Siha, NHEK‐CaSki and NHEK‐HaCaT are shown in Figures 6 and 7. The . test statistics used did not identify any bands for the combination HaCaT‐SiHa, for either standard or modulated Raman. The lack of statistically significant spectral differences here explain the reduced classification accuracy for the 4 cell line validation, where the largest populations of misidentified cells were from the HaCaT‐SiHa pairing. A lack of significant spectral differences may suggest that mechanisms behind the biological similarities between these 2 cell lines (eg, their ability to proliferate indefinitely) make a greater contribution to the Raman spectra than those characteristics which constitute a difference (such as DNA content). Significance bands for HaCaT‐CaSki and SiHa‐CaSki were only seen for the modulated Raman dataset, again suggesting that the application of PCA to differential spectra provides more useful information than traditional approaches. Prior to consideration of significance bands, it can be seen in the standard Raman spectra that for each comparison of NHEKs to the 3 established cell lines, the same peaks are demonstrating significant change. The peak at approximately 1590 cm^−1^ (adenine/guanine) only appears for the established cell lines, although significant difference is seen only for NHEK‐HaCaT here. It is interesting that the largest peak at approximately 1450 cm^−1^ (CH2/CH3 deformation of lipids/collagen/other hydrocarbon modes) is significant for NHEK‐HaCaT (normal vs immortalised) but not for the standard normal vs. transformed cell types (NHEK against SiHa or CaSki). The modulated NHEK‐CaSki comparison shows several significant spectral differences not seen in the remaining comparisons. The statistically significant bands and tentative peak assignments based on previous literature 9, 24 are shown in Appendix S2, Table 2.

Overall, it is clear that the use of a differential spectrum in modulated Raman spectroscopy highlights different statistically significant regions of the spectrum in comparison to standard Raman spectra. Due to the format of the differential spectrum, real Raman peaks correspond to differential zero‐crossing points. Therefore differential spectral variation represents subtle alterations in the shape, slope and position of peak ‘shoulders’ as opposed to absolute peak height and position. Small variations in these parameters result from changes to the population of a molecular group (in the case where multiple Raman peaks occupy the same region) or changes to the environment of a bond. This may lead to a significant increase in utility for the differential spectrum approach in biological contexts, as Raman peaks frequently overlap and biological changes are often the result of alterations in molecular conformation in addition to type (as is the case for protein conformation or DNA packaging). The only regions which were statistically significant for all standard Raman spectral comparisons were those corresponding to Amide I:*β*‐sheet/C‐O stretch/*ν*(C‐C) and Amide III:*α*‐helix/collagen/CH in phospholipids/C‐C in fatty acids. It is suggested that the increased density of protein conformational changes may arise from histones 9. Many of the remaining significant peak differences also result from DNA‐associated structures, including DNA O‐P‐O backbone stretching and polynucleotide chains. The only differential region which demonstrated statistically significant variations in each case was that of the symmetric ring breathing mode of phenylalanine and tryptophan.

### 3.3 Impact of intracellular variation

**Figure 8:**
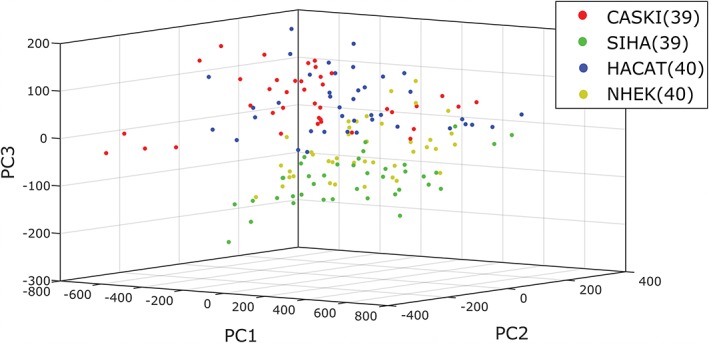
The PCA plot for the overall classification of cell lines without consideration of location

**Figure 9:**
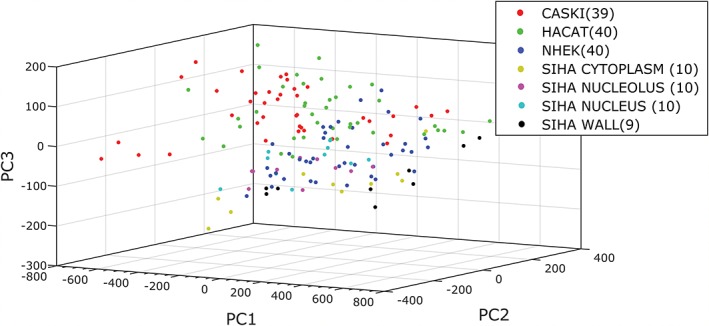
The PCA plot for classification where SiHa cells have been considered in terms of sampling by nucleus, nucleolus, cytoplasm or cell wall. The remaining location‐expanded plots can be found in Appendix S3

It was previously found that the Raman spectra for intracellular locations in CHO‐K1 cells were very distinct, with the nucleus providing the optimum signal‐to‐noise ratio 12. However, that data was generated from adherent cells grown directly onto glass slides, giving the characteristic flattened morphology associated with cells in culture. This distinctive morphology did not translate to the spectral acquisitions from fixed cells considered in this article: the cell rounding effects associated with trypsinisation and fixing made the consistent identification of intracellular organelles challenging. Typical brightfield images are shown in Appendix S3, Figure 1. To investigate the importance of intracellular location on characterisation, further samples of the Section 3.2 cell lines were fixed and each cell interrogated multiple times at locations designated as cell wall, cytoplasm, nucleus and nucleolus. Typical images are shown in Appendix S3, Figure 2. Cells were rejected if the specified components could not be discerned clearly. The nucleolus was identified as a high‐density sphere within the nuclear volume. All 4 datasets were recorded in the modulated setup only and analysed as discussed previously. Figure 8 shows the overall PCA plot from the cells, disregarding laser spot location. The resulting confusion matrix and classifications are detailed in Appendix S3, Table 3. Despite some misclassifications (particularly for NHEKs), the average predictive accuracy is high at 91.2%. The analysis can then be expanded to consider location for each cell line in turn. This can be seen for the SiHa line in Figure 9—the remaining expanded PCA plots can be seen in Appendix S3, Figures 3–5. The classification accuracies for the location‐specific subset of each expanded matrix are summarised in Table 3. These are much lower and more variable than both classifications by cell line and location‐based results from de Luca et al. experiment 12, suggesting that there is insufficient spectral disparity to separate clusters by location when rounded cells are interrogated in solution.

**Table 3:** The percentage classification accuracies for the cell lines by intracellular location. The average classification error is a single SD from the mean

Loc.	Classification accuracy (%)				Average
	NHEK	SiHa	CaSki	HaCaT	
Nucleus	50	60	50	60	55 ± 5
Nucleolus	50	20	50	80	50 ± 21
Cytoplasm	44	88	44	80	64 ± 20
Cell wall	60	50	60	80	63 ± 11

As such, the matrices were then considered by grouping points according to their location inside (nucleus, nucleolus) or outside (cytoplasm, cell wall) the nucleus. The summarised classification accuracies shown in Table 4. The resulting accuracies from this test indicate that there is sufficient spectral difference to separate these location clusters from inside and outside the nucleus with an accuracy approaching that of cell line classification. However, the fixed cells do not provide sufficient spectral difference to account for specific cellular regions with the accuracy seen for adherent cells interrogated directly on their culture substrates. Thus, cell shape and sampling method should be a key consideration for sampling location accuracy.

**Table 4:** The percentage classification accuracies for the cell lines based on location in reference to the nucleus. The average classification error is a single SD from the mean

Loc.	Classification accuracy (%)				Average
	NHEK	SiHa	CaSki	HaCaT	
Inside	93.3	90.0	90.0	94.1	91.9 ± 1.9
Outside	88.9	86.7	77.8	100.0	88.4 ± 7.9

### 3.4 Impact of other variation sources

**Figure 10:**
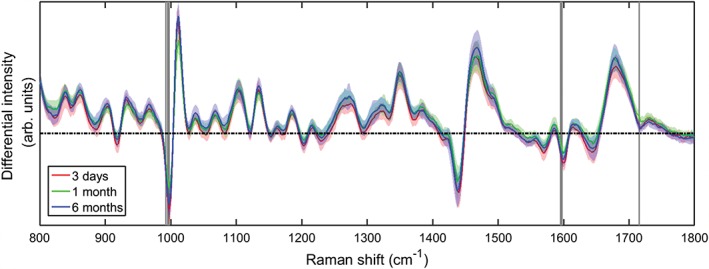
Average spectra of 50 cells from each sample, stored in PreservCyt for 3 days, 1 month or 6 months

**Figure 11:**
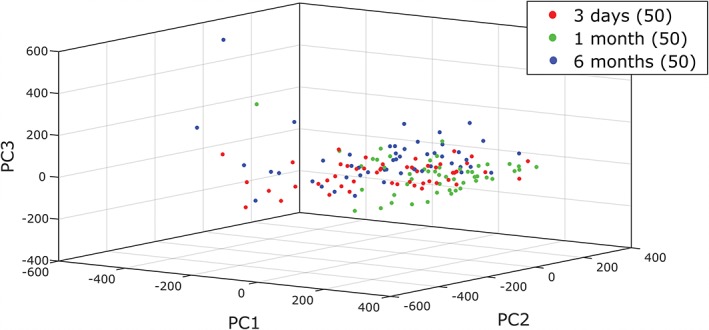
The PCA plot of the 3 ‘archived’ fixed samples, stored for different periods of time

Finally, the potential effects of storage time on spectral classification were investigated. Recent work by Hobro et al. considered spectral differences between live mouse embryonic fibroblast cells and those fixed with different agents, including methanol 25. Methanol acts as both a fixing and permeabilisatio n agent which dehydrates cells and precipitates proteins. In that case, a significant loss of overall Raman signal was observed in cells fixed with methanol, which were mostly associated with lipid bands. The effects of storage in fixative were not considered by Hobro et al. Based on the routine morphological and genomic analysis of stored smear samples without alterations to data, we hypothesised that changes over time after initial fixation would not be significant. If such changes do occur, it would have considerable impact on the use of Raman spectroscopy as a tool for clinical sample classification where samples may be archived in fixing solution for long periods of time either before analysis, for revisiting borderline samples or for comparing with new samples for disease progression. A total of 150 cells were interrogated from SiHa cell samples which had been stored at −20°C for 3 days, 1 month or 6 months in PreservCyt before following the same protocols described previously. The average Raman spectra are in Figure 10. Pairwise comparisons of each sample type as conducted before revealed significant differences between 3 days and 1 month cells, and between 1 and 6 months cells, but not between the 1 day and 6 months cells. These differences occur in both the former cases at 992 to 997 cm^−1^ (ring breathing of tryptophan/phenylalanine), 1595 cm^−1^ (adenine/guanine) and 1715 cm^−1^ (C=O, lipids). None of these bands were indicated in the live‐fixed cell comparison for methanol 25. Figure 11 shows the corresponding PCA plot, where grouping can be seen clearly for the 3 days and 6 months clusters in the PC1/PC2 and PC2/PC3 projections. The confusion matrix (Table 5) for the 3 days and 6 months samples are consistent with the hypothesis that no changes should occur during storage, as classification accuracy is low. However, the 1 month sample shows high‐classification accuracy, which indicates that there are sufficient spectral differences to classify these nominally identical cells in relation to the remaining samples. The lack of trend according to storage time suggests that while storage should not be an issue for classification, other sources of variation are present and can be detected by the WMRS.

**Table 5:** The confusion matrix resulting from cross‐validation of the fixed cell storage time dataset, with accompanying percentage classification accuracy

Actual	Predicted			Class acc. (%)
	3 d	1 mo	6 mo	
3 d	29	0	21	58.0
1 mo	1	47	2	94.0
6 mo	17	1	32	64.0

## 4 DISCUSSION

In Sections 3.1 and 3.2, the comparisons of standard and modulated methods for spectral acquisition clearly demonstrate that WMRS is able to provide superior cell classification accuracy. The ability to discriminate between HPV positive and negative cervical carcinoma cell lines is an encouraging step towards the potential integration of HPV and smear testing using optical discrimination. However, it must be noted that these carcinoma lines represent different mechanisms of origin, and by extension molecular pathologies. The ability to also identify HPV status in normal cervical cells would be a requirement of an integrated platform and so this will require consideration. The presence of significant spectral differences only in the modulated Raman acquisition between SiHa and CaSki cells, which represent primary and metastatic cervical carcinoma respectively, also points towards greater clinical utility for the sampling method and resulting spectral analysis. The investigation of location impact for spectral sampling provides important data regarding the expected differences between sampling particular cell morphologies or methods of collection. It is suggested that the lower specificity of sampling location for fixed cells in suspension compared to previous experiments with adherent, plated cells is due to the superposition of spectral data from different components of the cell, as the surface area occupied by the cell is lower. This lower margin of error could provide benefits for coupling the spectrometer with automated cell sampling capabilities, and will have to be taken into consideration for clinical samples acquired with a cytology brush. Finally, although overall cell classification was unaffected by storage time in fixative, the relationship between spectral characteristics and fixation was complex, consistent with recent evidence that methanol fixatives affect spectral data, particularly in relation to lipid peaks 25. The sensitivity of the technique to these changes suggests that similar issues with sample heterogeneity will have to be addressed with large clinical datasets.

## 5 CONCLUSION

In this study, fluorescence‐free wavelength modulated Raman spectroscopy has been used to consider the benefits and practicalities of such a system in the context of label‐free classification of fixed cell samples, such as those from cervical screening. The use of wavelength modulation in Raman spectroscopy and the resulting removal of autofluorescence provides clear benefits to its ability to automatically classify cells over conventional Raman scattering, without the requirement for manual baseline fitting, additional lasers or optical gates. It is entirely feasible that the technique could be combined with other imaging methods such as optical coherence tomography 26 or digital holographic microscopy 27 in order to provide a cell ‘topography’ map for automated point sampling. It is hoped that such a system could be introduced to clinical settings in order to reduce the burden on manual cytological diagnosis, as well as increasing the likelihood of a correct outcome for patients.

## Supporting information


**Appendix S1.** Details of the origin and cell culture reagents.
**Appendix S2.** Statistically significant Raman spectral regions.
**Appendix S3.** Confusion matrix and classification accuracy

## References

[1] CRUK , Screening, http://www.cancerresearchuk.org/about‐cancer/cervical‐cancer/getting‐diagnosed/screening. (accessed: 14 April 2017).

[2] CRUK , 2012 Review of the UK breast screening programme, http://www.cancerresearchuk.org/about‐cancer/breast‐cancer/screening/screening‐2012‐review. (accessed: 14 April 2017).

[3] J. H. Smith , J. Patnick , Achievable Standards, Benchmarks for Reporting, and Criteria for Evaluating Cervical Cytopathology, 3rd ed., NHS Cancer Screening Programmes, Sheffield 2013.

[4] P. D. Palma , P. G. Rossi , G. Collina , A. M. Buccoliero , B. Ghiringhello , M. Lestani , G. Onnis , D. Aldovini , G. Galanti , G. P. Casadei , M. Aldi , V. Gomes , P. Giubilato , G. Ronco , and The NTCC pathology group, Am. J. Clin. Pathol. 2008, 129, 75. 18089491 10.1309/EWYGWFRRM8798U5P

[5] A. L. Mitchell , K. B. Gajjar , G. Theophilou , F. L. Martin , P. L. Martin‐Hirsch , J. Biophotonics 2014, 7, 153. 24648213 10.1002/jbio.201400018

[6] H. J. Butler , L. Ashton , B. Bird , G. Cinque , K. Curtis , J. Dorney , K. Esmonde‐White , N. J. Fullwood , B. Gardner , P. L. Martin‐Hirsch , M. J. Walsh , M. R. McAinsh , N. Stone , F. L. Martin , Nat. Protoc. 2016, 11, 664. 26963630 10.1038/nprot.2016.036

[7] D. Lin , S. Feng , J. Pan , Y. Chen , J. Lin , G. Chen , S. Xie , H. Zeng , R. Chen , Opt. Express 2011, 19, 13565. 21747512 10.1364/OE.19.013565

[8] P. R. T. Jess , M. Mazilu , K. Dholakia , A. C. Riches , C. S. Herrington , Int. J. Cancer 2009, 124, 376. 18942712 10.1002/ijc.23953

[9] P. R. T. Jess , D. D. W. Smith , M. Mazilu , K. Dholakia , A. C. Riches , C. S. Herrington , Int. J. Cancer 2007, 121, 2723. 17724716 10.1002/ijc.23046

[10] I. R. M. Ramos , A. Malkin , F. M. Lyng , Biomed. Res. Int. 2015, 2015, 561242. 26180802 10.1155/2015/561242 PMC4477184

[11] E. Vargis , E. M. Kanter , S. K. Majumder , M. D. Keller , R. B. Beaven , G. G. Rao , A. Mahadevan‐Jansen , Analyst 2011, 136, 2981. 21666910 10.1039/c0an01020k

[12] A. C. De Luca , M. Mazilu , A. Riches , C. S. Herrington , K. Dholakia , Anal. Chem. 2010, 82, 738. 20017474 10.1021/ac9026737

[13] D. Wei , S. Chen , Q. Liu , Appl. Spectrosc. Rev. 2015, 50, 387.

[14] B. B. Praveen , M. Mazilu , R. F. Marchington , C. S. Herrington , A. Riches , K. Dholakia , PLoS ONE 2013, 8, e67211. 23825643 10.1371/journal.pone.0067211 PMC3692494

[15] M. Mazilu , A. C. De Luca , A. Riches , C. S. Herrington , K. Dholakia , Opt. Express 2010, 18, 11382. 20588999 10.1364/OE.18.011382

[16] M. Chen , N. McReynolds , E. C. Campbell , M. Mazilu , J. Barbosa , K. Dholakia , S. J. Powis , PLoS ONE 2015, 10, e0125158. 25992777 10.1371/journal.pone.0125158 PMC4439084

[17] E. Canetta , M. Mazilu , A. C. De Luca , A. E. Carruthers , K. Dholakia , S. Neilson , H. Sargeant , T. Briscoe , C. S. Herrington , A. C. Riches , J. Biomed. Opt. 2011, 16, 037002. 21456875 10.1117/1.3556722

[18] H. Abdi , L. J. Williams , WIREs Comp. Stat. 2010, 2, 433.

[19] B. M. Wise , N. L. Ricker , D. F. Veltkamp , B. R. Kowalski , *Process Control and Quality* 1990, 1, 41.

[20] R. Bhatia , The Scottish HPV Archive (report), http://www.ed.ac.uk/pathology/research/scottish-hpv-archive. (accessed: 14 April 2017).

[21] P. Boukamp , R. T. Petrussevska , D. Breitkreutz , J. Hornung , A. Markham , N. E. Fusenig , J. Cell Biol. 1988, 106, 761. 2450098 10.1083/jcb.106.3.761 PMC2115116

[22] F. Friedl , I. Kimura , T. Osato , Y. Ito , Proc. Soc. Exp. Biol. Med. 1970, 135, 543. 5529598 10.3181/00379727-135-35091a

[23] R. A. Pattillo , R. O. Hussa , M. T. Story , A. C. Ruckert , M. R. Shalaby , R. F. Mattingly , Science 1977, 196, 1456. 867042 10.1126/science.867042

[24] A. C. S. Talari , Z. Movasaghi , S. Rehman , I. ur Rehman , Appl. Spectrosc. Rev. 2015, 50, 46.

[25] A. J. Hobro , N. I. Smith , Vib Spectrosc 2017, 91, 31.

[26] M. Chen , J. Mas , L. H. Forbes , M. R. Andrews , K. Dholakia , J. Biophotonics, 2017, e201700129.

[27] N. McReynolds , F. G. M. Cooke , M. Chen , S. J. Powis , K. Dholakia , Sci. Rep. 2017, 7, 43631. 28256551 10.1038/srep43631 PMC5335250

