# Supplementary material for: Towards automated cancer screening: Label‐free classification of fixed cell samples using wavelength modulated Raman spectroscopy
Source: J Biophotonics. 2018 Jan 30;11(4):e201700244. doi: 10.1002/jbio.201700244 (PMC6540043; doi:10.1002/jbio.201700244)
Supplement: Supplementary file 1 — Appendix S1. Details of the origin and cell culture reagents. Appendix S2. Statistically significant Raman spectral regions. Appendix S3. Confusion matrix and classification accuracy [file JBIO-11-na-s001.docx]

**Supplementary Information S1**

Details of the origin and cell culture reagents used for each cell type are shown in Table 1.

Table 1 Cultured cell lines and supporting reagents. Reagents were purchased from Gibco unless otherwise stated. HaCaT cells were kindly donated by Dr Duncan Sproul at the Institute of Genetics and Molecular Medicine, Edinburgh.

| Cultured cell lines | | | |
| --- | --- | --- | --- |
| Cell line name | Culture medium | Disassociation agent | Cell provenance |
| NHEKs (purchased from Lonza) | KGM-Gold (Lonza), 0.1% hydrocortisone, 0.1% transferrin, 0.05% epinephrine, 0.1% GA-1000, 0.4% BPE, 0.1% rhEGF, 0.1% insulin | Trypsin/EDTA 0.25% (Lonza) | Primary keratinocytes, neonatal foreskin |
| SiHa (purchased from ATCC) | EMEM (ATCC), 10% [v/v] FBS (Labtech), 1% [v/v] Penicillin-Streptomycin | Trypsin/EDTA 0.05% | HPV16 +ive squamous cell carcinoma, adult cervix |
| CaSki (purchased from ATCC) | RPMI-1640, 10% [v/v] FBS (Labtech), 1% [v/v] Penicillin-Streptomycin | Trypsin/EDTA 0.25% | HPV16 +ive squamous cell carcinoma, adult cervix (metastasis to small intestine) |
| HaCaT (acquired from IGMM) | DMEM, 10% [v/v] FBS (Labtech), 1% [v/v] Penicillin-Streptomycin | Trypsin/EDTA 0.25% | Immortalised keratinocytes, adult skin |

All cell stocks were maintained in 10cm tissue culture plates (Greiner Bio-One) and incubated at $37^{\circ}\text{C}$ in 5% CO_2_. The three established cell lines (HaCaT, SiHa, CaSki) were subcultured 2-3 times per week dependent on seeding density (typically 1:3 to 1:10 dilution ratio). NHEKs were subcultured 1-2 times per fortnight due to slow growth, with 50% media replacement on alternating days.

To subculture the established cell lines, the culture medium was removed and the cells washed once using 2ml sterilised PBS. 2ml of the appropriate disassociation agent was added and the cells returned to the incubator for 5 minutes. 8ml of appropriate medium was added to neutralise the disassociation reagent and dislodge the cells. New tissue culture plates were prepared with the appropriate type and volume of medium according to the dilution ratio used, to a total plate volume of 10ml. Both media and disassociation reagent were warmed to $37^{\circ}\text{C}$ in a water bath prior to use.

For NHEK subculturing, the supernatant was removed and plates were washed with 5ml of HEPES BSS. 2ml of 0.25% Trypsin/EDTA at room temperature was added and the cells returned to the incubator for three minutes. The plates viewed at 4x brightﬁeld microscope magniﬁcation until 90% of cells were disassociated. The trypsinisation process was neutralised using 4ml room temperature TNS. 4ml of KGM-Gold medium was added and the cells added to new plates (1:2 to 1:5 dilution ratio) containing KGM-Gold medium up to 10ml. The KGM-Gold/Trypsin inhibitor mix was replaced with fresh KGM-Gold the following day following cell adherence.

Supplementary Information S2

Table 2 Statistically signiﬁcant Raman spectral regions for classiﬁcation. Statistically signiﬁcant Raman spectral regions for used for classiﬁcation of cell lines. Vibrational modes are assigned based on previous literature (Jess et al. 2007, Talari et al. 2014). Regions shown in bold represent those for which the NHEK signature was higher than that of the comparator. Regions shown in italics refer to peak shoulders rather than the peak itself (shown in normal text).

| Signiﬁcant Raman spectral regions (cm^−1^) | | | | | | |
| --- | --- | --- | --- | --- | --- | --- |
| Vibrational mode | NHEK-SiHa (s) | NHEK-CaSki (s) | NHEK-HaCaT (s) | NHEK-SiHa (m) | NHEK-CaSki (m) | NHEK-HaCaT (m) |
| DNA O-P-O backbone stretching |  |  |  |  | **800** |  |
| Ring breathing in tyrosine/C-C stretching in proline/DNA O-P-O backbone stretching |  |  |  |  | **837-842, 856-866** | ***844*** |
| C-C skeletal stretching in protein |  |  |  |  | **931-936** | **934** |
| Symmetric ring breathing mode of phenylalanine/tryptophan ring breathing |  | 1004 |  | *1016-1018* | 999-1023 | *1014-1021* |
| *ν*(C-N)/DNA O-P-O backbone stretching/C-C amino side chains |  | 1092-1096 | 1101-1103 |  | 1103-1119 | 1110-1117 |
| C-C skeletal stretching |  |  |  |  | 1138-1145 |  |
| Cytosine, guanine, adenine/antisymmetric phosphate vibrations |  |  |  |  | *1192-1197* |  |
| Amide III: *α*-helix & collagen, CH in phospholipids, C-C in fatty acids | 1230-1264 | 1220-1274 | 1237-1267, *1269-1291* |  | 1269 | *1267-1269* |
| Polynucleotide chain (DNA bases) | 1332-1354, *1362-1378* | 1324-1371 |  |  | *1352-1366* |  |
| CH_2_ deformation in lipids/adenine/cytosine/C-O symmetric stretch |  |  | *1328-1392* |  | 1387-1401 |  |
| CH2/CH3 deformation of lipids & collagen/CH2 wagging/CH2/CH3 deformation |  |  | *1490-1495* |  | 1448-1469 *1448-1469* | ***1436*** |
| Adenine/guanine |  |  | 1596 |  |  |  |
| Amide I: *β*-sheet/C-O stretch/*ν*(C-C) trans, lipids, fatty acids | *1680-1692* | *1681-1683* | *1680-1686* |  | 1685-1720, 1685-1720 | 1699-1709 |
| C-O lipids |  |  |  |  | 1725 |  |

Supplementary Information S3

Table 3 confusion matrix resulting from cross-validation of the intracellular location dataset before consideration of internal location. The average classiﬁcation accuracy is 91.2%.

|  | Predicted | | | |  |
| --- | --- | --- | --- | --- | --- |
| Actual | CaSki | SiHa | HaCaT | NHEK | Class acc. (%) |
| CaSki | 38 | 0 | 0 | 1 | 97.4 |
| SiHa | 0 | 36 | 2 | 1 | 92.3 |
| HaCaT | 1 | 0 | 37 | 2 | 92.5 |
| NHEK | 0 | 2 | 5 | 33 | 82.5 |

The confusion matrix and classiﬁcation accuracy for the intracellular location data prior to consideration of location are shown in Table 3.

Examples of cell type and positions used for location-base sampling are shown in Figures 1 and 2.

The location-based PCA classiﬁcation plots for NHEK, HaCaT and CaSki are shown below.

Figure 1 Typical brightﬁeld images of the ﬁxed cells from each type used for analysis in Sections 3.2 and 3.3. Brightﬁeld images were recorded with a 4x oil-immersion objective. Cells are typically 10*μ*m in diameter.

Figure 2 Typical brightﬁeld images of the ﬁxed cells used for analysis, with the position of the laser spot shown for each of the sampling locations.

Figure 3 The PCA plot for classiﬁcation where NHEK cells have been considered in terms of sampling by nucleus, nucleolus, cytoplasm or cell wall.

Figure 4 The PCA plot for classiﬁcation where HaCaT cells have been considered in terms of sampling by nucleus, nucleolus, cytoplasm or cell wall.

Figure 5 The PCA plot for classiﬁcation where CaSki cells have been considered in terms of sampling by nucleus, nucleolus, cytoplasm or cell wall.
